# Neutral effect of Zishen Yutai Pill on frozen-thawed embryo transfer: a propensity score matching study

**DOI:** 10.3389/fendo.2024.1379590

**Published:** 2024-08-29

**Authors:** Xiaolian Yang, Jiali Cai, Li Jiang, Xiaoming Jiang, Zhenfang Liu, Jinghua Chen, Kaijie Chen, Chao Yang, Jie Geng, Caihui Ma, Jianzhi Ren, Lanlan Liu

**Affiliations:** ^1^ Reproductive Medicine Center, The Affiliated Chenggong Hospital of Xiamen University, Xiamen, Fujian, China; ^2^ Medical College, Xiamen University, Xiamen, Fujian, China

**Keywords:** IVF-FET, Zishen Yutai pill, live birth rate, endometrial preparation, corpus luteum

## Abstract

**Objective:**

To investigate whether using Zishen Yutai Pills (ZYP) following embryo transfer would affect the live birth rate in frozen-thawed embryo transfer (FET) cycles.

**Methods:**

A retrospective analysis was performed on 15044 FET cycles in the Reproductive Medicine Center of The Affiliated Chenggong Hospital of Xiamen University from January 2013 to December 2020. Patients who used Zishen Yutai Pills were defined as Zishen Yutai Pills Group (ZYP, n=2735), while patients who did not use them were defined as Non- Zishen Yutai Pills Group (Non-ZYP, n=12309). The propensity score matching method was used to control for potential confounders between the two groups, and logistic regression analysis was also used to assess whether using ZYP would affect the live birth rate.

**Results:**

After propensity score matching, basic characteristics were similar between the two groups. Using ZYP did not increase the pregnancy rate (51.5% vs. 52.7%, P=0.372), and live birth rate (43.0% vs. 44.7%, P=0.354). This was also confirmed by the logistic regression analysis results (OR=0.95, 95%CI=0.85-1.06). In the subgroup analysis of the endometrial preparation protocols, however, it was found that the use of ZYP in patients with natural cycles increased the live birth rate (47.4% vs. 41.5%, P=0.004). A significant interaction between endometrial preparation and ZYP was found (OR=1.38, 95%CI=1.07-1.79) in the multivariate model.

**Conclusion:**

The use of ZYP may not improve the live birth rate of unselected patients in FET cycles. However, a future study is needed on the effect of ZYP in natural cycles for endometrial preparation.

## Introduction

Assisted reproductive technologies (ART) provide an important option for infertility treatment, but they are also expensive, complex, and not without significant risk of failure (1). Many adjuvants and supplements were used in the procedure of ART and infertility treatment in an attempt to optimize the pregnancy outcome or improve the live birth rate (2). Herbal therapy represents an important aspect of the use of adjuvants and supplements in infertility treatment. A recent survey suggested that over two-thirds of women seeking infertility used herbal therapy previously and over half of women used herbal medicine and supplement currently (3). In China and East Asia, Chinese herbal medicine (CHM) has been used to treat various diseases including infertility, and is reported to enhance the success of live births in infertile couples treated with ART (4–7), possibly by improving oocyte quality, ovarian function, and endometrial receptivity (8). However, the evidence remained biased and heterogeneous, warranting further investigations to justify the use of CHMs in ART treatment.

Zishen Yutai Pill (ZYP) is one of the few representative CHMs that are supported by RCT evidence (9–11). A recent meta-analysis of RCTs suggested that ZYP may improve clinical symptoms such as human chorionic gonadotropin, progesterone, estradiol, duration of abdominal pain, duration of vaginal bleeding, and fibrinogen in women with threatened miscarriage (12). For patients receiving ART treatment, a rate ratio of 1.14 (95% CI 1.00–1.30) for clinical pregnancy in comparison with placebo treatment was reported when ZYP was administrated from the preceding menstrual cycle of the fresh embryo transfer (13). These data suggested that ZYP may play a role in complementary treatment in ART. However, it is still less known whether the use of ZYP would benefit ART-related clinical scenarios other than fresh embryo transfer, such as patients receiving frozen embryo transfer (FET).

FET is an important scenario of ART treatment. In the past decade, with the improvement of cryopreservation technology and increasing demand to reduce ovarian hyperstimulation syndrome, promote single embryo transfer, optimize endometrial receptivity, and preserve fertility, more and more patients choose FET (14). There is still a lack of evidence supporting the use of ZYP in FET cycles. The present study aims to investigate whether ZYP has an impact on live birth rates in people with FET cycles. In this study, 15044 FET cycles were included, of which 2735 cycles used ZYP. A propensity score matching method was used to account for comparability between the two groups by balancing the biases and confounders, and binary logistic regression was carried out to confirm the findings.

## Materials and methods

Institutional review board approval for this study was obtained from The Affiliated Chenggong Hospital of Xiamen University. Informed consent was not necessary because this retrospective research was based on non-identifiable records.

### Study subjects

This retrospective study was performed at the reproductive medicine center of The Affiliated Chenggong Hospital of Xiamen University. Patients who underwent FET cycles from 2013 to 2020 were enrolled and retrospectively analyzed. Exclusion criteria:(a) cycles with missing or incomplete data, (b) patients with uterine factors like uterine adhesion, uterine autonomy, and previous cesarean scar diverticulum (PCSD). A total of 15,044 FET cycles were included, of which 2,735 cycles used ZYP. Cycles using ZYP were defined as the ZYP group and cycles without ZYP were defined as the non-ZYP group.

### Embryo vitrification and thawing

Ovarian stimulation protocols, embryo culture procedures, and vitrification protocol used for embryo cryopreservation were all as we previously described (15), and embryo thawing was carried out using the corresponding thawing kit. Embryos were placed in the blastocyst culture medium (K-SIBM, Cook) and cultured in an incubator at 37°C with 6% CO_2_ until transfer. Survival of thawed embryos was assessed under an inverted microscope depending on whether blastocysts showed severely damaged cellular content or not.

### Endometrial preparation and embryo transfer

The natural cycle (NC) or modified natural cycle (mNC), the hormone replacement therapy (HRT) cycle, and the HRT cycle in combination with gonadotropin-releasing hormone agonist (GnRHa) downregulation were used for endometrial preparation.

In NC cycles, transvaginal ultrasonography was used to track follicle growth from cycle days 9 to 11. Luteinizing hormone and estradiol levels were checked every three days once the leading follicle’s diameter reached 14 mm. The day when a spontaneous LH surge was noted was considered as the ovulation day (day 0) and luteal phase support was started on the same day. Blastocyst transfer was scheduled for the fifth day after ovulation and cleavage period embryo transfer was scheduled for the third day after ovulation. In cases of modified NC cycles, patients were given 6500 units of HCG on the day of the LH surge and an intramuscular progesterone injection of 40 mg/day was also started on the same day, the second day after the LH surge was considered as ovulation day (day 0) and embryo transfer was scheduled.

In HRT cycles, administration of oral estradiol valerate was carried out using a daily dose of 6 mg between cycle days 1 and 14. As soon as the endometrial thickness reached 7-8 mm (designated as day 0), a progesterone injection (40 mg) was administered.

In GnRHa-HRT cycles, GnRHa was injected on the first day of the menstrual cycle, and estrogen administration was initiated on the first day of the second menstrual cycle.

For all cycles, embryo transfers were carried out on days 3 or 5 under transabdominal ultrasound guidance using a Guardia Access Embryo Transfer catheter (K-JETS-7019-SIVF, Cook, IN, USA). If there was no available blastocyst on day 5, and day 6 transfers were scheduled. Up to week ten of pregnancy, luteal support was maintained. Patients with an elevated hCG level underwent an ultrasound examination 28 days after embryo transfer, and the gestational sac was determined to be a clinical pregnancy.

### ZYP medication

ZYP (Guangzhou BaiyunshanZhongyi Pharmaceutical Co., Ltd., Sinopharm Z44020008, Specifications: 5g/bag, 6 bags/box) was used on the day of embryo transfer in FET cycles, 3 times/day, 5g/time, until the 14th day after embryo transfer when the biochemical pregnancy test was carried out. The treatment continued for 3 months if the pregnancy was confirmed. The daily dosage adheres to the manufacturer`s instructions (https://www.gz111.com/product/41.htm) and previous studies (13, 16). Because there were no clear indications for the use of ZYP in FET cycles by the time of the study, the assignment of patients was based on the preference of the clinicians.

### Statistical analysis

The primary outcome was the live birth rate (LBR). Secondary outcomes of the study included clinical pregnancy rate (PR) and miscarriage rate (MR).

To minimize the effect of confounders and covariates, the outcomes were evaluated in both a propensity score (PS) matched cohort and an unmatched cohort. The selection of covariates was based on experience and existing knowledge with the guidance of a directed acyclic graph with DAGitty software (**Supplementary Figure S1**). Potential confounders and selection biases were accounted for by propensity score matching (17).

Propensity scores were calculated using logistic regression based on potential variables related to the prognosis (18). The variables included age, Body Mass Index (BMI), basal endocrine parameters (FSH, LH, and AFC), previous maternal history, infertility-related diagnoses (including polycystic ovary syndrome [PCOS], history of intrauterine adhesion separation, endometriosis, and tubal factor), previous embryo transfer attempts, endometrial preparation protocols, oocyte yield, insemination protocols, E_2_ level, and endometrial thickness measured on the day of ovulation, ultrasonic types of the endometrium, the number and quality of the transferred embryo, the day of transfer. A one-to-one nearest neighbor matching method without substitution is performed to match data between group ZYP and non-ZYP with a caliper width equal to 0.03 (19). To investigate whether differences in medication regimens between ZYP and non-ZYP groups and the day of embryo transfer affected live birth, the two groups were stratified and PS was matched separately for each subgroup. Standard differences (D) were calculated to evaluate the balance of the distribution of the baseline characteristics between the two groups before and after PS matching using a cobalt package. D < 0.1 was used as the threshold to indicate a negligible difference in the mean or prevalence of a covariate between exposure groups. The distribution of propensity scores was also identical after matching (**Supplementary Figure S2**).

Multivariate logistic regression was used to confirm the outcome of PS matching. A multivariate logistic analysis was carried out following matching in an attempt to adjust for potential residual confoundings. We also used binary logistic regression analysis to assess the association between ZYP and live birth and adjusted for important covariates and potential confounders.

Continuous variables are represented by medians (first quartile, third quartile), while categorical variables are represented as n (%). Continuous variables were analyzed using the Wilcoxon test, and categorical variables were analyzed using the Chi-square test or Fisher’s exact test, P <0.05 was considered to be significant. All analyses were performed in R.

## Results

### Comparison of baseline data between ZYP group and Non-ZYP group before and after PSM

A total of 15,044 patients were collected, including 12,309 cases in the non-ZYP group and 2,735 cases in the ZYP group. As shown in **Table 1**, there were significant differences in the data before matching, including the female age, male age, female BMI, parity, PCOS, history of intrauterine adhesion separation, oocyte yield, E_2_ level measured on the day of ovulation, ultrasonic types of the endometrium, order of ET, endometrial preparation protocols, stage of transferred embryos (P<0.05). After PS matching, there were 2733 cases in the ZYP group and 2733 cases in the non-ZYP group, and there was no significant statistical significance in all indicators (D<0.1). Distributions of the PSs before and after PS matching were shown in **Supplementary Material** (**Supplementary Figure S2**).

**Table 1:** Comparison of baseline characteristics between ZYP group and Non-ZYP group before and after PSM.

Variables	Before matching		*P*	*D	After matching		*P*	*D
	non-ZYP	ZYP			NON-ZYP	ZYP		
	(N=12309)	(N=2735)			(N=2733)	(N=2733)		
Female age, year	31.0 [28.0,34.0]	32.0 [29.0,35.0]	<0.001	0.1991	32.0 [29.0,35.0]	32.0 [29.0,35.0]	0.896	0.0036
Male age, year	33.0 [30.0,36.0]	33.0 [30.0,37.0]	<0.001	0.1201	33.0 [30.0,37.0]	33.0 [30.0,37.0]	0.896	-0.0035
Female BMI, kg/m^2^	20.8 [19.3,22.5]	21.0 [19.5,22.6]	0.0223	0.0475	20.9 [19.5,22.6]	21.0 [19.5,22.6]	0.656	0.0119
Male BMI, kg/m^2^	23.7 [21.5,25.9]	23.5 [21.3,25.7]	0.105	-0.0345	23.5 [21.4,25.7]	23.5 [21.3,25.7]	0.995	0.0002
Basal FSH, mlU/ml	6.68 [5.73,7.90]	6.80 [5.75,8.08]	0.224	0.0304	6.79 [5.79,8.07]	6.80 [5.75,8.08]	0.901	0.0043
Basal LH, mlU/ml	4.49 [3.35,6.02]	4.49 [3.35,6.01]	0.308	-0.0131	4.38 [3.31,5.87]	4.49 [3.35,6.00]	0.0531	0.0047
Antral follicle count	11.0 [8.00,15.0]	11.0 [8.00,15.0]	0.145	0.031	11.0 [8.00,16.0]	11.0 [8.00,15.0]	0.979	-0.0007
Parity>=1	2077 (16.9%)	528 (19.3%)	0.0026	0.0243	533 (19.5%)	527 (19.3%)	0.864	0.0022
Gravidity			0.0696				0.671	
0	6191 (50.3%)	1321(48.3%)		-0.02	1276 (46.7%)	1321(48.3%)		0.0165
1	3159 (25.7%)	710 (26.0%)		0.003	729 (26.7%)	709 (25.9%)		-0.0073
2	1702 (13.8%)	405 (14.8%)		0.0098	435 (15.9%)	404 (14.8%)		-0.0113
3	789 (6.4%)	169 (6.2%)		-0.0023	162 (5.9%)	169 (6.2%)		0.0026
>3	468 (3.8%)	130 (4.8%)		0.0095	131 (4.8%)	130 (4.8%)		-0.0004
Diagnosis								
Endometriosis	1093 (8.9%)	235 (8.6%)	0.659	-0.0029	235 (8.6%)	235 (8.6%)	>0.99	<0.001
Tubal factor	8034 (65.3%)	1779(65.0%)	0.842	-0.0022	1732 (63.4%)	1777(65.0%)	0.214	0.0165
PCOs	1055 (8.6%)	186 (6.8%)	0.00265	-0.0177	180 (6.6%)	186 (6.8%)	0.787	0.0022
History of intrauterine adhesion separation	439 (3.6%)	155 (5.7%)	<0.001	0.021	152 (5.6%)	154 (5.6%)	0.953	0.0007
Oocyte yield	12.0 [8.00,17.0]	11.0 [7.00,15.0]	<0.001	-0.2038	11.0 [8.00,15.0]	11.0 [7.00,15.0]	0.797	-0.0066
Insemination protocols			0.928				0.914	
ICSI	2604 (21.2%)	594 (21.7%)		0.0056	584 (21.4%)	592 (21.7%)		0.0029
IVF	9012 (73.2%)	1990(72.8%)		-0.0045	2003 (73.3%)	1990(72.8%)		-0.0048
RICSI	691 (5.6%)	151 (5.5%)		-0.0009	146 (5.3%)	151 (5.5%)		0.0018
E_2_ measured on the day of ovulation, pg/ml	297[213,420]	296[213,414]	<0.001	-0.1921	284[206,397]	295 [213,413]	0.898	-0.0026
Endometrial thickness on the day of ovulation, mm	8.60 [7.70,9.90]	8.70 [7.70,10.0]	0.0996	0.0349	8.70 [7.70,9.90]	8.70 [7.70,10.0]	0.647	0.0125
Ultrasonic types of endometrium			<0.001				0.595	
A	263 (2.1%)	36 (1.3%)		-0.0082	31 (1.1%)	36 (1.3%)		0.0018
B	11049(89.8%)	2527(92.4%)		0.0263	2544 (93.1%)	2525 (92.4%)		-0.007
C	997 (8.1%)	172 (6.3%)		-0.0181	158 (5.8%)	172 (6.3%)		0.0051
ET order			<0.001				0.706	
1	3795 (30.8%)	600 (21.9%)		-0.0889	625 (22.9%)	600 (22.0%)		-0.0091
2	5626 (45.7%)	1234(45.1%)		-0.0059	1212 (44.3%)	1234(45.2%)		0.008
3	1832 (14.9%)	544 (19.9%)		0.0501	524 (19.2%)	543 (19.9%)		0.007
4	1056 (8.6%)	357 (13.1%)		0.0447	372 (13.6%)	356 (13.0%)		-0.0059
Endometrial preparations			<0.001				>0.99	
GnRHa +HRT	2642 (21.5%)	910 (33.3%)		0.1181	913 (33.4%)	908 (33.2%)		-0.0018
HRT	3821 (31.0%)	545 (19.9%)		-0.1112	548 (20.1%)	545 (19.9%)		-0.0011
Modified NC	346 (2.8%)	66 (2.4%)		-0.004	65 (2.4%)	66 (2.4%)		0.0004
NC	5468 (44.4%)	1201(43.9%)		-0.0051	1194 (43.7%)	1201(43.9%)		0.0026
Others	32 (0.3%)	13 (0.5%)		0.0022	13 (0.5%)	13 (0.5%)		<0.001
Stage of transferred embryos			<0.001				0.742	
Day 3	2699 (21.9%)	360 (13.2%)		-0.0876	339 (12.4%)	360 (13.2%)		0.0077
Day 5	8181 (66.5%)	1885(68.9%)		0.0246	1914 (70.0%)	1884(68.9%)		-0.011
Day 5 + 6	83 (0.7%)	69 (2.5%)		0.0185	62 (2.3%)	69 (2.5%)		0.0026
Day 6	1342 (10.9%)	421 (15.4%)		0.0449	418 (15.3%)	420 (15.4%)		0.0007

Data were presented as median [first quartile, third quartile] for continuous variables and n (percentage) for categorical variables. *D: Standardized difference. The absolute value of D is less than 0.1, cohorts can be considered to be balanced with respect to the demographics being assessed. OPU, oocyte pick up; PCOs, polycystic ovarian syndrome; BMI, body mass index; FSH, follicle-stimulating hormone; LH, luteinizing hormone; E_2_, Estradiol; ET, Embryo transfer; GNRHa, gonadotropin agonist; HRT, hormone replacement therapy; NC, natural cycle.

### Comparison of pregnancy outcomes between ZYP and non-ZYP groups before and after PSM

Results in **Table 2** showed that administration of ZYP does not appear to affect clinical outcomes. Before matching, pregnancy rates [52.7% vs 53.8%, RR=0.98, (95% CI 0.94-1.02)], live birth rates [44.3% vs 45.5%, RR=0.97, (95%CI 0.93-1.02)], ectopic rates [0.8% vs 1.0%, RR=0.8, (95%CI 0.43-1.47)], and malformation rates [0.6% vs 1.1%, RR=0.53, (95% CI, 0.24-1.16)] were slightly lower in ZYP group, and the early miscarriage rate (15.0% vs 14.5%) was slightly higher than that in the non-ZYP group, but there was no significant difference (P>0.05). After matching, the pregnancy rates [52.7%vs51.5%, RR=1.02, (95% CI, 0.97-1.08)], live birth rates [44.7%vs43.0%, RR=1.03, (95% CI, 0.97-1.09)], ectopic pregnancy rates [0.8%vs0.7%, RR=1.17, (95% CI, 0.51-2.71)], and late miscarriage rates [2.2%vs2.1%, RR=1.04, (95% CI, 0.64-1.71)] were higher in the ZYP group, and the early miscarriage rates [12.8%vs13.4%, RR=0.96, (95% CI, 0.8-1.16)], and malformation rates [0.6%vs1.2%, RR=0.49, (95% CI, 0.2-1.2)] were slightly lower than those in the non-ZYP group. None of the differences were statistically significant (P>0.05).

**Table 2:** Outcomes of transfer cycles between ZYP and non-ZYP groups before and after PSM.

Outcomes	Before matching		P	After matching		P
	NON ZYP	ZYP		NON ZYP	ZYP	
Pregnancy			0.304			0.372
Rate,%	6626/12309	1442/2735		1408/2735	1441/2735	
	(53.8%)	(52.7%)		(51.5%)	(52.7%)	
RR(95% CI)	ref	0.98(0.94,1.02)		Ref	1.02(0.97,1.08)	
Live birth			0.25			0.354
Rate,%	5602/12309	1211/2735		1176/2735	1210/2735	
	(45.5%)	(44.3%)		(43%)	(44.7%)	
RR(95% CI)	ref	0.97(0.93,1.02)		Ref	1.03(0.97,1.09)	
Ectopic			0.52			0.709
Rate,%	69/6626	12/1442		10/1408	12/1441	
	(1%)	(0.8%)		(0.7%)	(0.7%)	
RR(95% CI)	ref	0.8(0.43,1.47)		Ref	1.17(0.51,2.71)	
Miscarriage						
Early			0.821			0.912
Rate,%	797/6226	185/1442		188/1408	185/1441	
	(12%)	(12.8%)		(13.4%)	(12.8%)	
RR(95% CI)	ref	1.07(0.92,1.24)		Ref	0.96(0.8,1.16)	
Late						
Rate,%	153/6226	32/1442		30/1408	32/1441	
	(2.5%)	(2.2%)		(2.1%)	(2.2%)	
RR (95% CI)	ref	0.96(0.66,1.4)		Ref	1.04(0.64,1.71)	
Malformation			0.223			0.11
Rate,%	61/5602	7/1211		14/1176	7/1210	
	(1.1%)	(0.6%)		(1.2%)	(0.6%)	
RR (95% CI)	ref	0.53(0.24,1.16)		Ref	0.49(0.2,1.2)	

RR, Ratio rate; CI, confidence index.

### Summary of binary logistic regression analysis results

The binary logistic regression analysis with whether or not live birth was the dependent variable, and the Hosmer-Lemeshow test of the final model showed that the model fit was good. The adjusted OR value of the pre-matching ZYP group for the non-ZYP group was 1.27 (95% CI=0.94~1.70). After matching, the OR value was 0.95 (95% CI=0.85~1.06), the regression coefficient was -0.049 (P=0.391), and the difference was not significant. Results were shown in **Table 3**.

**Table 3:** Summary of binary logistic regression analysis results.

Characteristic	Before matching			After matching		
	OR	95%CI	P	OR	95%CI	P
ZYP	1	0.91-1.10	>0.9	1.06	0.95-1.18	0.3
Female age, year	0.96	0.95-0.97	<0.001	0.96	0.94-0.99	0.001
Male age, year	1	0.99-1.01	0.5	0.98	0.96-1.00	0.051
Female BMI, kg/m^2^	1.01	0.99-1.02	0.4	1.02	0.99-1.05	0.3
Male BMI, kg/m^2^	1	0.99-1.0	0.6	1	0.98-1.02	>0.9
Basal FSH, mlU/ml	0.98	0.97-1.00	0.044	0.98	0.95-1.00	0.092
Basal LH, mlU/ml	1.01	1.00-1.02	0.11	1.03	1.01-1.05	0.002
Antral follicle count	1.01	1.00-1.02	0.002	1.01	0.99-1.02	0.3
Parity						
0	ref	ref	ref	ref	ref	ref
1	0.93	0.83-1.03	0.2	0.96	0.81-1.15	0.7
Gravidity						
0	ref	ref	ref	ref	ref	ref
1	1	0.91-1.09	>0.9	1.02	0.88-1.18	0.8
2	1	0.90-1.12	>0.9	1	0.83-1.21	>0.9
3	0.88	0.75-1.02	0.1	0.8	0.61-1.05	0.1
>3	1.02	0.84-1.23	0.9	0.95	0.70-1.28	0.7
Endometriosis	0.91	0.80-1.03	0.12	0.85	0.69-1.04	0.12
Tubal factor	1.11	1.02-1.20	0.014	1.09	0.96-1.25	0.2
PCOs	1.02	0.88-1.18	0.8	0.94	0.73-1.23	0.7
History of intrauterine adhesion separation						
	0.88	0.73-1.05	0.15	0.86	0.67-1.12	0.3
Oocyte yield	1.01	1.01-1.02	<0.001	1.03	1.03-1.04	<0.001
Insemination protocol						
ICSI	ref	ref	ref	ref	ref	ref
IVF	0.96	0.87-1.05	0.4	1.07	0.92-1.25	0.4
RICSI	0.92	0.79-1.08	0.3	0.91	0.70-1.20	0.5
E_2_ measured on the day of ovulation, pg/ml						
	1	1.00-1.00	0.9	1	1.00-1.00	0.3
P measured on the day of ovulation, ng/ml						
	1.25	0.83-1.89	0.277	1.08	0.96-1.22	0.203
Endometrial thickness measured on the day of ovulation, mm						
	1.08	1.06-1.10	<0.001	1.06	1.02-1.09	0.001
Ultrasonic types of endometrium						
A	ref	ref	ref	ref	ref	ref
B	1.45	1.13-1.86	0.004	1.26	0.75-2.16	0.4
C	1.21	0.92-1.59	0.2	1.22	0.69-2.18	0.5
ET order						
1	ref	ref	ref	ref	ref	ref
2	0.87	0.80-0.95	0.001	1	0.86-1.16	>0.99
3	0.78	0.70-0.87	<0.001	0.92	0.76-1.10	0.4
4	0.79	0.68-0.90	<0.001	0.84	0.68-1.04	0.12
Endometrial preparations						
GnRHa+HRT	ref	ref	ref	ref	ref	ref
HRT	0.67	0.61-0.74	<0.001	0.57	0.48-0.68	<0.001
Modified NC	0.86	0.68-11.07	0.2	0.82	0.56-1.20	0.3
NC	0.92	0.79-1.07	0.3	0.88	0.7-1.12	0.3
stage of transferred embryos						
Day 3	ref	ref	ref	ref	ref	ref
Day 5	1.76	1.46-2.14	<0.001	1.91	1.72-2.13	<0.001
Day 5 + 6	2.86	1.91-4.28	<0.001	3.22	2.28-4.57	<0.001
Day 6	1.09	0.86-1.37	0.5	1.17	1.02-1.34	0.024

BMI, Body mass index; FSH, follicle-stimulating hormone; LH, luteinizing hormone; E_2_, Estradiol; ET, Embryo transfer; PCOs, Poly Cystic Ovary Syndrome; E_2,_ Estradiol; P, Progesterone; ET, Embryo transfer; GNRHa, gonadotropin agonist; HRT, hormone replacement therapy; NC, natural cycle.

### Endometrial preparation protocols of subgroup analysis

When stratified according to endometrial preparation protocols, an increase in live birth rates in natural cycle patients was associated with ZYP use (P<0.05). In NC cycles, the OR that described the association between live birth and ZYP treatment increased was 1.14 times (RR = 1.14, 95% Cl 1.04-1.25) than that in the GnRH-HRT cycles, indicating that the live birth rate in the ZYP group was 1.14 times that of the non-group. The use of ZYP benefited more natural cycle patients, resulting in a 5.9% increase in live birth rates (41.5% vs. 47.4%). The association between other endometrial preparations and live birth rate was not significant. Results were shown in **Table 4**.

**Table 4:** Outcomes of ZYP and non-ZYP stratified by endometrial preparation after PSM.

		Live birth	P	RR (95% CI)	crude OR (95% CI)	adjusted OR (95%CI)^a^	ratio of OR
GnRHa			0.177				
	non-ZYP	433/913(47.4%)		Ref	ref	ref	ref
	ZYP	401/908 (44.2%)		0.93(0.84,1.03)	0.88(0.73,1.05)	0.89(0.73,1.09)	ref
NC			0.004				
	non-ZYP	495/1194 (41.5%)		Ref	ref	ref	ref
	ZYP	569/1201 (47.4%)		1.14(1.04,1.25)	1.27(1.08,1.49)	1.24(1.05,1.48)	1.38(1.07,1.79)
mNC			0.671				
	non-ZYP	29/65 (44.6%)		Ref	ref	ref	ref
	ZYP	27/66(40.9%)		0.92(0.62,1.36)	0.94(0.74,1.2)	0.98(0.75,1.27)	1.05(0.77,1.45)
HRT			0.801				
	non-ZYP	216/548 (39.4%)		Ref	ref	ref	ref
	ZYP	207/545(38.0%)		0.96(0.83,1.12)	0.86(0.43,1.72)	0.93(0.3,2.87)	0.85(0.4,1.8)

a: adjusted by age, BMI, basal FSH, LH, AFC, previous maternal history, PCOs, history of intrauterine adhesion separation, endometriosis, tubal factor, previous embryo transfer attempts, endometrial preparation protocols, oocyte yield, insemination protocols, E_2_ and endometrial thickness measured on the day of ovulation, ultrasonic types of endometrium, the number and quality of the transferred embryo, the day of transferred.

## Discussion

Similar to many of its CHM counterparts, ZYP is considered to be a multi-functional adjuvant therapy for reproductive medicine and infertility treatment (20). Its role may include a curative effect for luteal dysfunction, miscarriage, and recurrent spontaneous abortion, which may be relevant to embryo implantation and development following transfer. These effects may theoretically benefit the patients receiving FET. In the present study, we found a neutral effect of administrating ZYP following embryo transfer in FET cycles. The evidence may not support the routine use of ZYP in FET cycles as an adjuvant during early luteal phase support. However, for patients receiving natural cycle endometrial preparation, a positive association between ZYP administration and live birth was found.

Previous studies showed that the positive effects of ZYP treatment on IVF outcomes are exclusively based on fresh embryo transfer cycles, and the duration of ZYP treatment is much longer than the present study. In fresh cycles, patients generally start using ZYP before oocyte retrieval. For instance, in the study of Chen et al. (13), patients receiving the gonadotropin-releasing hormone analogs long protocol, and both the ZYP and placebo were administered orally three times per day in a dose of 5 g from the day of down-regulation. For patients receiving GnRH antagonist protocol, both the ZYP and placebo were administered orally three times per day in a dose of 5 g from day 19-23 in a previous cycle until the day of the pregnancy test (2 weeks after ET). Patients with long protocol, also oral ZYP from the date of down-regulation, 5 g/time, 3 times/d until 35 days after embryo transfer in Bai’s study (21). The early start of ZYP treatment allows the adjuvant to target several reproduction-related processes of the cycle, including follicular development, endometrial growth, and embryo implantation. Clinical observations showed that ZYP may benefit the patient by increasing the oocyte yield and availability of good-quality embryos (22). Therefore, in fresh cycles, the multiple roles of ZYP were difficult to figure out due to the subsequential embryo transfer following oocyte development. In contrast, FET separates the oocyte collection and embryo transfer in different cycles. As the availability of the embryos is determined by previous ovarian stimulation cycles, the role of ZYP in FET cycles is limited to supporting endometrial growth and embryo implantation, allowing an evaluation of the effect of ZYP on these processes. Although our data did not support a generalized benefit of ZYP treatment following embryo transfer, the role of ZYP administration during endometrial preparation could not be excluded, as the treatment may be associated with a higher endometrial thickness (23).

The supposed effect on oocyte development might play a key role in the pharmacological mechanisms of ZYP during the IVF procedure. A previous study have found the expression of bone morphogenetic protein 15 (BMP15) and growth differentiation factor 9 (GDP9) in the follicular fluid (FF), these two factors are paracrine regulators for oocyte development and are very important for its development (24). Both BMP15 and GDF9 stimulate follicle growth and granule cell proliferation (25). Furthermore, the expression of these two factors is mediated by the phosphatidylinositol 3 kinase/protein kinase B (PI3K/AKT) pathway (26). A metabolomics study found that the content of PI3K/AKT-related metabolites could be upregulated in patients taking ZYP (11). Therefore, ZYP upregulates the expression of BMP15 and GDF9 in FF by changing the PI3K/AKT content, thereby improving oocyte quality (27). However, molecular targets such as PI3K/AKT may also be involved in endometrial growth and embryo implantation (28). A similar mechanism may also affect the endometrium and improve the endometrial receptivity (29). Although the majority of the previous studies focused on the potential effects of ZYP on ovaries and endometrium, the complexity of herbal materials made a difficult to study the synergistic effects of traditional Chinese medicine (TCM) prescriptions. Like other types of traditional medicine, TCM is expected to affect the biological system rather than the specific molecular targets (30). A recent metabonomics study suggested that blood concentrations of metabolites such as tauroursodeoxycholic acid, L-asparagine, L-glutamic acid, kynurenic acid, 11-deoxycorticosterone, melatonin glucuronide, and hydroxytyrosol, indicating changes in lipid and amino acid metabolism (11). It may suggest a wider mechanism of ZYP actions. To better understand the mechanism and target of therapy, the investigators and manufacturers should provide a more detailed picture of how the therapy affects each targeted organ and process.

A stratified analysis of endometrial preparation regimens showed that the live birth rate was significantly higher in the ZYP group than in the non-ZYP group (41.5% vs. 47.4%) for NC patients. Endometrial preparation is an integral part of FET, and the most commonly used regimens are NC and HRT (31). The HRT regimens use exogenous estradiol and progesterone to support endometrial growth and differentiation, lacking a normal follicular dynamic and corpus luteum which are present in the NC cycles. The corpus luteum is associated with endometrial development, and luteal insufficiency can lead to endometrial dysplasia and may impair the role of ZYP in improving implantation support in normal pregnancy (32). The ovary is an active endocrine and paracrine organ that controls the pregnancy beyond generating the female gametes, and the ovary may be the target of ZYP (20). Analyses of the blood components in ZYP-treated animals may suggest that the mechanism of effects may include neuroactive ligand-receptor interaction and steroid hormone biosynthesis, and these effects might be weakened when the luteal function is insufficient (33).

On the one hand, a study revealed that the HRT cycle has a substantial impact on placental development compared with the modified natural cycles (mNC), resulting in a higher incidence of preeclampsia, postpartum hemorrhage, and Cesarean section (34). Another study showed that exogenous estrogen and progesterone administration in artificial cycles may interfere with the readiness of the endometrium (35). It has been shown that ZYP can improve the outcomes of a fresh cycle by upregulating the expression of the homologous frame gene A10 (HOXA10) and improving the uterine receptivity impaired by ovarian stimulation (9). To benefit the patients with HRT, the timing of ZYP administration may be earlier than the day following ET, possibly covering the entire cycle period as what has been done in an ovarian stimulation cycle.

The main advantage of this study is that we have substantially large sample sizes of data compared to previous studies. There are also some limitations because it is a retrospective study, confounded by unknown or unmeasured factors. The results are also possibly biased. Since there was a lack of clear indications, the patient assignment may be a significant source of bias. For instance, the patients who chose to receive ZYP may have a poorer prognosis or be less confident, seeking adjuvants for solutions to uncertainty. Nevertheless, we use the PSM method to correct potential conjuring factors as much as possible to minimize possible bias. On the other hand, however, the differences in endometrial preparation may hint at a potential selection bias. The observed interaction between endometrial preparation and ZYP treatment could also be explained by unmeasured confounding in different populations.

The belief in traditional medicine is common in patients seeking fertility treatment. According to a large cross-sectional survey, traditional Chinese medicine is the top fertility treatment option for infertile couples (29.4%) (36). The belief in the effects of TCM may also be a potential motive driving patients to receive ZYP in FET cycles. The beliefs of patients may also lead to potential bias and confounding in several aspects. The patients who intend to receive ZYP may be biased toward those with longer disease courses, higher desire, or better informed concerning traditional medicine. According to a previous review, patients who have stronger beliefs in the effectiveness of TCM beliefs may be less likely to adhere to self-management and medication (11). It could suggest unknown or unmeasured confounding to the outcomes. For instance, these patients may also seek other TCM adjuvants outside the ART program. The patients` belief in efficacy may be also associated with a placebo effect (37). Since the study was retrospective, the placebo effect is also a reason for caution.

In conclusion, our study suggests that ZYP may not be a routine adjuvant in FET cycles. However, the positive association between live birth rate and ZYP treatment in NC cycles warrants further investigation into the subgroups of patients.

## Data Availability

The raw data supporting the conclusions of this article will be made available by the authors, without undue reservation.
