# Supplementary material for: Neutral effect of Zishen Yutai Pill on frozen-thawed embryo transfer: a propensity score matching study
Source: Front Endocrinol (Lausanne). 2024 Aug 29;15:1379590. doi: 10.3389/fendo.2024.1379590 (PMC11390590; doi:10.3389/fendo.2024.1379590)
Supplement: Supplementary file 1 [file DataSheet1.docx]

Supplementary Material


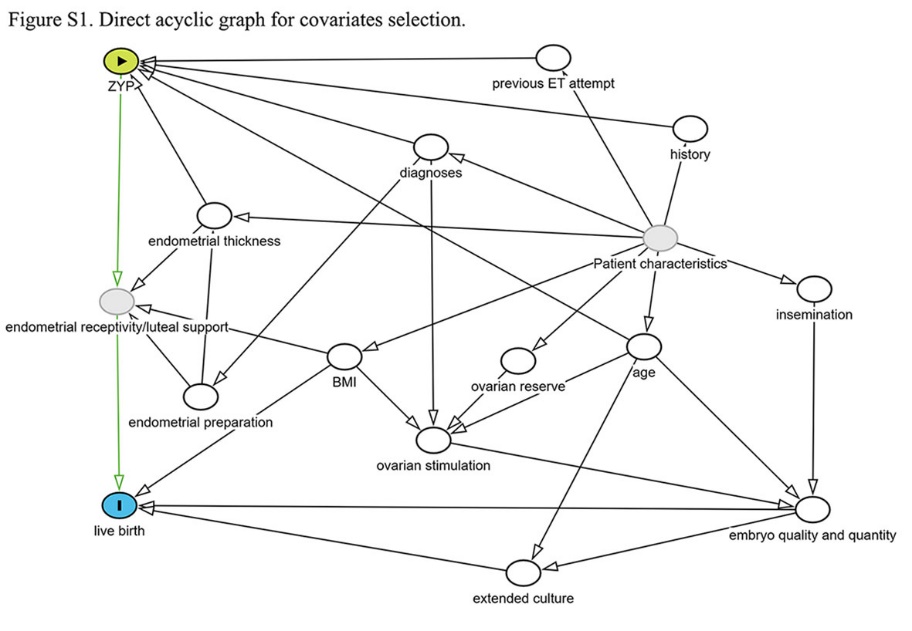


Figure S1. A DAGitty software was used for this directed acyclic graph, selection of covariates was based on experience and existing knowledge.


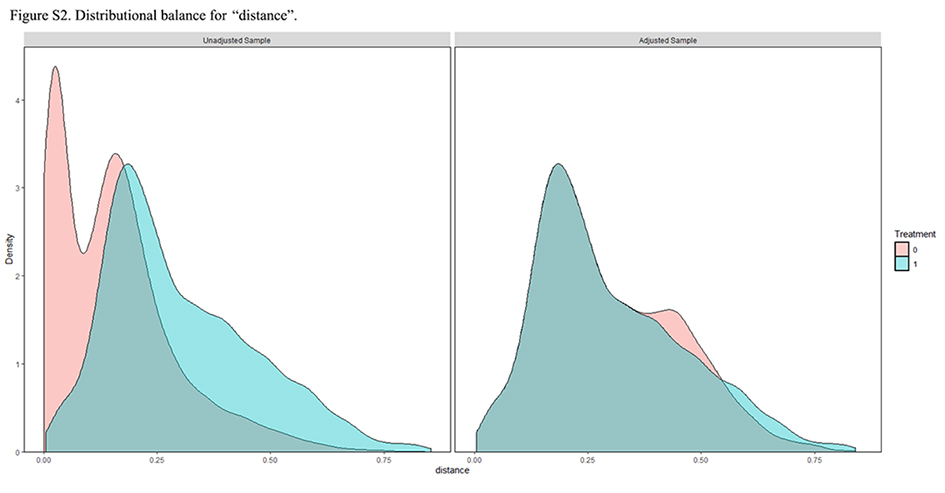


Figure S2. The matching was carried out with a MatchIt package，distributions of the PSs before and after PS matching were shown.
